# Artemisinin and partner drug resistance markers in *Plasmodium* *falciparum* from Tanzanian paediatric malaria patients, 2016–2022

**DOI:** 10.1186/s12936-025-05447-x

**Published:** 2025-07-01

**Authors:** Aveline Assey, Silvia Scialabba, Maria Mgella Zinga, Philip Koliopoulos, Welmoed van Loon, Caroline A. Minja, Britta Groendahl, Johannes Plett, Stephan Gehring, Mariam M. Mirambo, Neema Kayange, Frank P. Mockenhaupt, Stephen E. Mshana

**Affiliations:** 1 Department of Parasitology and Entomology, Catholic University of Health and Allied Sciences, P.O. Box 1464, Mwanza, Tanzania; 2 Centre of Paediatric and Adolescent Medicine, University Medical Center, 55131 Mainz, Germany; 3 Charité—Universitätsmedizin Berlin, Freie Universität Berlin and Humboldt-Universität Zu Berlin, Charité Center for Global Health, Institute of International Health, 13353 Berlin, Germany; 4 Department of Biochemistry and Molecular Biology, Catholic University of Health and Allied Sciences, P. O. Box 1464, Mwanza, Tanzania; 5 Department of Microbiology and Immunology, Catholic University of Health and Allied Sciences, P.O. Box 1464, Mwanza, Tanzania; 6 Department of Paediatrics, Catholic University of Health and Allied Sciences, P.O. Box 1464, Mwanza, Tanzania

**Keywords:** *Plasmodium falciparum*, *Pfmsp1*, *Pfmsp2*, Genetic diversity, Multiplicity of infection, *Pfk13*, Artemisinin resistance, *Pfmdr1*

## Abstract

**Background:**

*Plasmodium* *falciparum* malaria remains a significant public health concern in Tanzania, particularly among children under 5 years of age. The emergence and spread of partial artemisinin resistance in East Africa add to this concern. Specific mutations in the *P.* *falciparum* *kelch-13* (*Pfk13*) and *multidrug drug resistance 1* (*Pfmdr1*) genes are associated with artemisinin resistance and lumefantrine tolerance, respectively. The emergence of antimalarial drug resistance may be associated with unstable transmission in sub-Saharan Africa (SSA). Time-trends of *Pfk13* and *Pfmdr1* mutations as well as the multiplicity of infection (MOI) as a proxy for transmission intensity were investigated.

**Methods:**

Between 2016 and 2022, 173 *P. falciparum* PCR-positive samples were collected from febrile inpatient and outpatient children aged 3 months to 18 years at selected health facilities in Mwanza, Tanzania. *Pfk13* and *Pfmdr1* were amplified by PCR and Sanger-sequenced. Polymorphic *Pfmsp1* and *Pfmsp2* allelic markers were genotyped by nested PCR in 168 samples to assess MOI.

**Results:**

Among 143 samples successfully sequenced for *Pfk13*, 7.0% (10/143) exhibited non-synonymous mutations including the WHO-validated artemisinin resistance marker R561H in 1.4% (2 patients, 2022). As for *Pfmdr1*, the wild-type N86 allele was observed in 100% (97/97) of isolates, and about half (55/97) carried the wild-type Y184 allele. The mean multiplicity of infection (MOI) was 1.5, and did not change significantly over time. Single-genotype and polyclonal infections were observed in 59.3% (80/135), and 40.7% (55/135) respectively.

**Conclusion:**

This study from Mwanza, Tanzania demonstrates the presence of a validated artemisinin resistance marker *Pfk13* R561H in 2022 and suggests increased lumefantrine tolerance. MOI as a proxy marker of endemicity was low and stable over the six years of observation. The detection of these resistance markers reinforces the need for continuous genetic surveillance to sustain the efficacy of antimalarial therapies in paediatric patients.

## Background

The United Republic of Tanzania continues to experience a high malaria burden. The country ranked fifth in global malaria deaths in 2023, and is among the eight countries that saw a significant increase in cases in the past five years (almost 2 million). Malaria in Tanzania is predominantly caused by *Plasmodium falciparum*, and it affects mostly young children [1]. Artemisinin forms the cornerstone of artemisinin-based combination therapy (ACT), the global mainstay for falciparum malaria treatment, and artemisinin partial resistance (AR) is considered a major threat to malaria control in SSA [2]. Worryingly, the Kagera region of northwestern Tanzania was recently identified as one of the few places in sub-Saharan Africa (SSA) with confirmed artemisinin partial resistance (AR) [3]. While AR alone does not cause treatment failure and only has a mild clinical effect, i.e., delayed parasite clearance in patients treated with artemisinin-based combination therapy, AR sets the stage for the development of resistance to the ACT partner drugs [4]. In South-East Asia, such multi-resistant *P. falciparum* now causes high treatment failures in its epicentres [4, 5]. About 20 validated and candidate mutations in the *P. falciparum kelch-13* (*Pfk13*) gene have been associated with AR [6]. AR mutations observed in East Africa include 441L, 469Y/F, 561H, 622I, and 675 V [7]. True resistance markers for the major ACT partner drug lumefantrine have not been identified as of today, but variants in the *P. falciparum multidrug resistance 1* (*Pfmdr1*) gene are associated with increased tolerance [5, 8].

In 2021, molecular surveillance revealed a high prevalence of *Pfk13* 561H in the Kagera region in northwestern Tanzania [9], which linked with persisting day-3 parasitaemia after ACT [3]. The city of Mwanza, about 500 km from Kagera region by road, is also located in northwestern Tanzania, at the edge of a high transmission region that extends into Kagera [10]. Mwanza has a high population density, and forms an important transport hub between regions in East Africa.

The emergence of AR elsewhere in SSA has been hypothesized to be associated with interrupted malaria control or unstable transmission [3, 11]. In that context, the multiplicity of infection (MOI) of *P. falciparum* in patients can be used as a proxy indicator for malaria transmission in a region [12]. However, a direct correlation of MOI with the evolution of antimalarial resistance (AR) markers remains complex and is still under investigation [13, 14].

This study aimed to provide retrospective data on the presence of *Pfk13* and *Pfmdr1* markers in paediatric malaria patients in 2016–2022. In addition, data on MOI over the years are included to estimate potential changes of transmission intensity.

## Methods

### Study area

The study was conducted in Mwanza region at three healthcare facilities representing different levels of care: Bugando Medical Centre (Zonal and University Teaching Hospital), Sekou Toure (Regional Referral Hospital), Sengerema (Designated District Hospital CDH). Mwanza is located at the southern shore of Lake Victoria at an altitude of 1100 m and has approximately 3.7 million inhabitants. The region has a tropical/subtropical climate, an average temperature of 23.1 °C and an average rainfall of about 1000 mm/year [15].

### Study design

Between April 2016 and October 2022, 1116 children (3 months–18 years) with acute fever (> 38.0 °C) who met the criteria for presumptive malaria treatment according to World Health Organization (WHO) definition [16], were enrolled at the three above named health-care facilities. Informed consent was provided by parents or guardians and informed assent was signed by school-aged children. Whole blood was collected and tested with NADAL® Malaria Pf/Pan Ag 4 Species plus diagnostic test (nal von minden GmbH, Germany). Aliquots of whole blood were applied onto Whatman 903 Proteinsaver Cards (Cytiva, Germany), dried, archived as dried blood spots (DBS), and analysed in 2023. Clinical signs and symptoms, medication and discharge diagnosis were recorded for all the patients.

### Molecular analysis

Six punches were obtained from each DBS and resuspended in nuclease-free water, followed by incubation at 60 °C for 1 h with shaking at 500 rpm. The supernatant was collected and used for DNA extraction using the High Pure Viral Nucleic Acid Kit (Roche Diagnostics, Germany) following the manufacturer’s instructions, and stored at − 20˚C. In brief, the supernatant containing DBS resuspension is lysed using binding buffer and proteinase K. The released nucleic acids are bound to a glass fiber membrane in the High Pure Filter Tube, and washed to remove inhibitors and impurities. The purified nucleic acids are recovered using elution buffer. *Plasmodium* infection was confirmed using multiplex reverse transcriptase PCR combined with ELISA (multiplex-RT-PCR-ELISA) [17]. Positive samples were typed for *P. falciparum, Plasmodium vivax* and *Plasmodium malariae* using PCR [17].

To assess multiplicity of infection (MOI), nested PCR assays for the polymorphic *Pfmsp1* and *Pfmsp2* genes were done as previously described, with slight modifications [18, 19]. Briefly, sequences corresponding to the allele families of *P. falciparum msp1* block 2 (K1, Mad20, Ro33) and *msp2* block 3 (FC27, 3D7) were amplified. These alleles are characterized by conserved regions flanked by repetitive sequences of different lengths. Therefore, the size variation within the alleles can be used to distinguish different parasite clones by PCR fragment length polymorphism. Gel electrophoresis on 2% agarose was performed to assess amplicon size. As a reference for allele typing, genomic DNA from the *P. falciparum* laboratory strain NF54 (expressing the K1 and 3D7 alleles) was used as a positive control to verify PCR performance and amplicon size [18–20]. *Pfmsp1* and *Pfmsp2* alleles visible on the agarose gel were estimated for size and counted. Only fragments detected in the typical size range for *Pfmsp1* (150 to 300 bp) and *Pfmsp2* (200–500 bp) were considered. Fragments of an estimated size difference within 20 bp were considered the same allele [21]. The highest allele counts for either *Pfmsp1* or *Pfmsp2* defined MOI.

Regions of *Pfk13* and *Pfmdr1* were amplified by PCR assays as previously described (respectively, codons 440–680 and codons 1106–1298) [22, 23]. Amplicons were subsequently Sanger-sequenced (Eurofins Genomics, Germany), and aligned to 3D7 reference sequences (plasmoDB.org) using CodonCode Version 9.0.1.

### Data analysis

Mean MOI was calculated per year of sample collection, and its development over years was tested for trend using weighted linear regression. MOI between rural and urban health centres was compared using Welch’s t-test. Analysis was done in STATA version 15 and R version 4.4.2 [24, 25].

## Results

1116 febrile children were enrolled, and 15.5% (173) were *P. falciparum* positive by PCR, no other *Plasmodium* species were detected. Among these, the average temperature was 38.9 °C (range, 36.6–41.5), and mean age was 43.7 months (3–216). Male children slightly predominated (58.4%, 101/173). Microscopic examination was done on discretion of the clinician on 74 samples, of which 34 tested positive. Among the positive samples, parasite density results were available for 17. They had a geometric mean parasite density of 6,396 parasites/µL (95% confidence interval [CI], 2419–16,909).

### Diversity of *P. falciparum msp1* and *msp2* alleles and multiplicity of infection

**Fig. 1:**
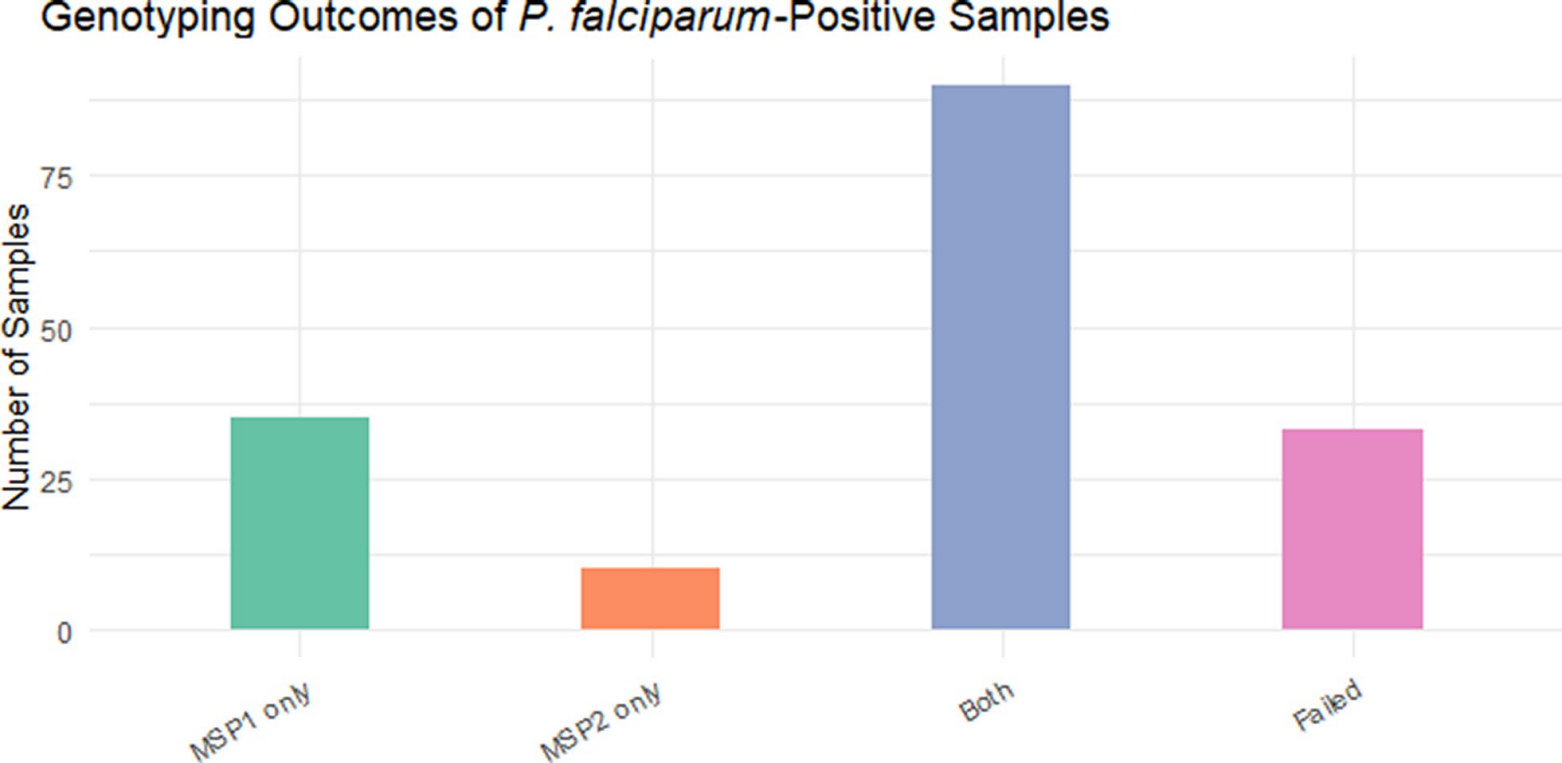
Genotyping outcomes of *P. falciparum*-positive samples. Bar chart showing the distribution of genotyping results: 20.8% of samples were positive only for *PfMSP1*, 6.0% only for *PfMSP2*, 53.6% for both markers, while 19.6% failed amplification

**Fig. 2:**
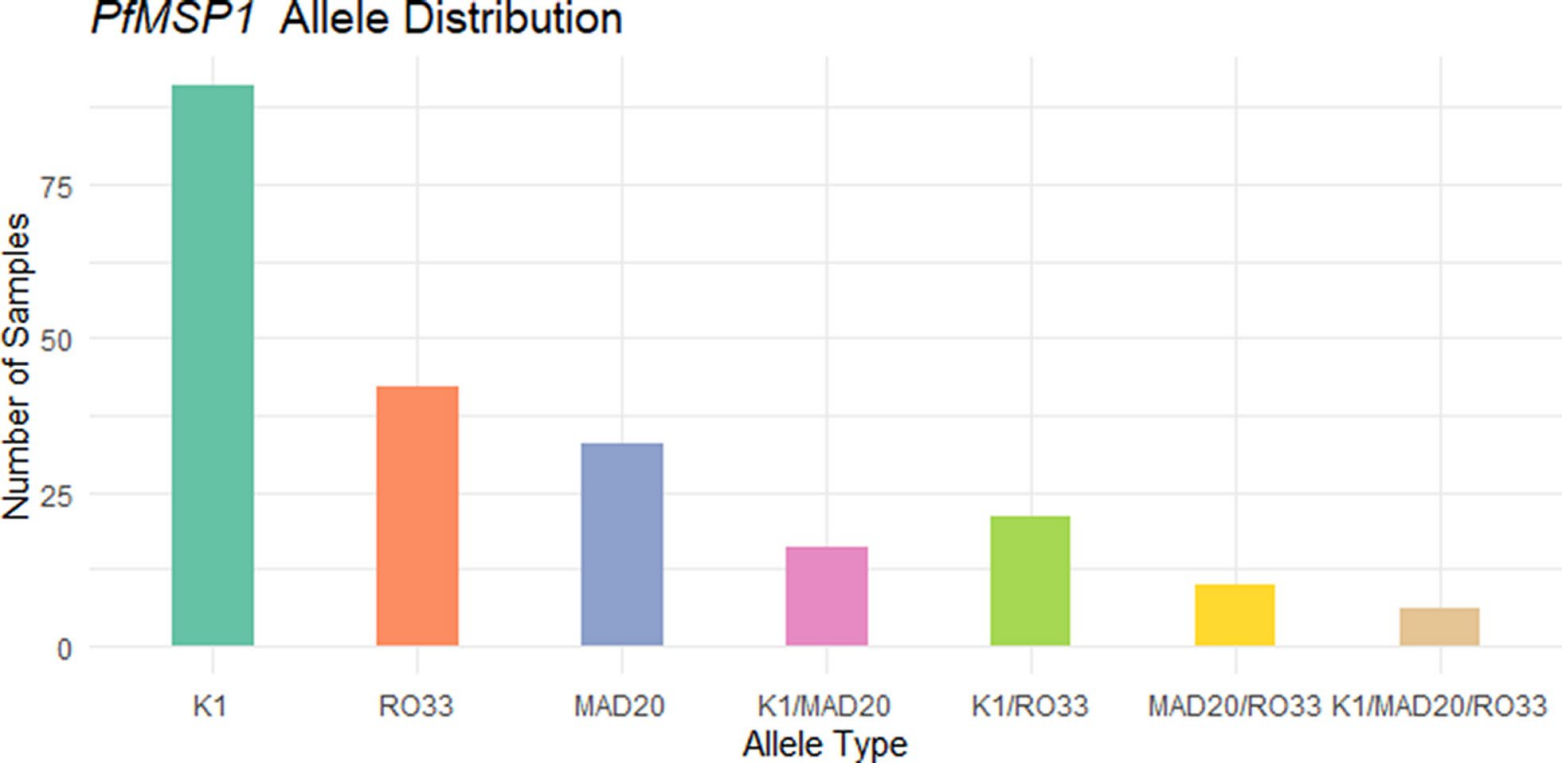
Distribution of *PfMSP1* allelic families. The K1 allele was the most prevalent (67.4%), followed by RO33 (31.1%) and MAD20 (24.4%). Mixed allele infections included K1/MAD20 (11.8%), K1/RO33 (15.5%), MAD20/RO33 (7.4%), and K1/MAD20/RO33 (4.4%)

**Fig. 3:**
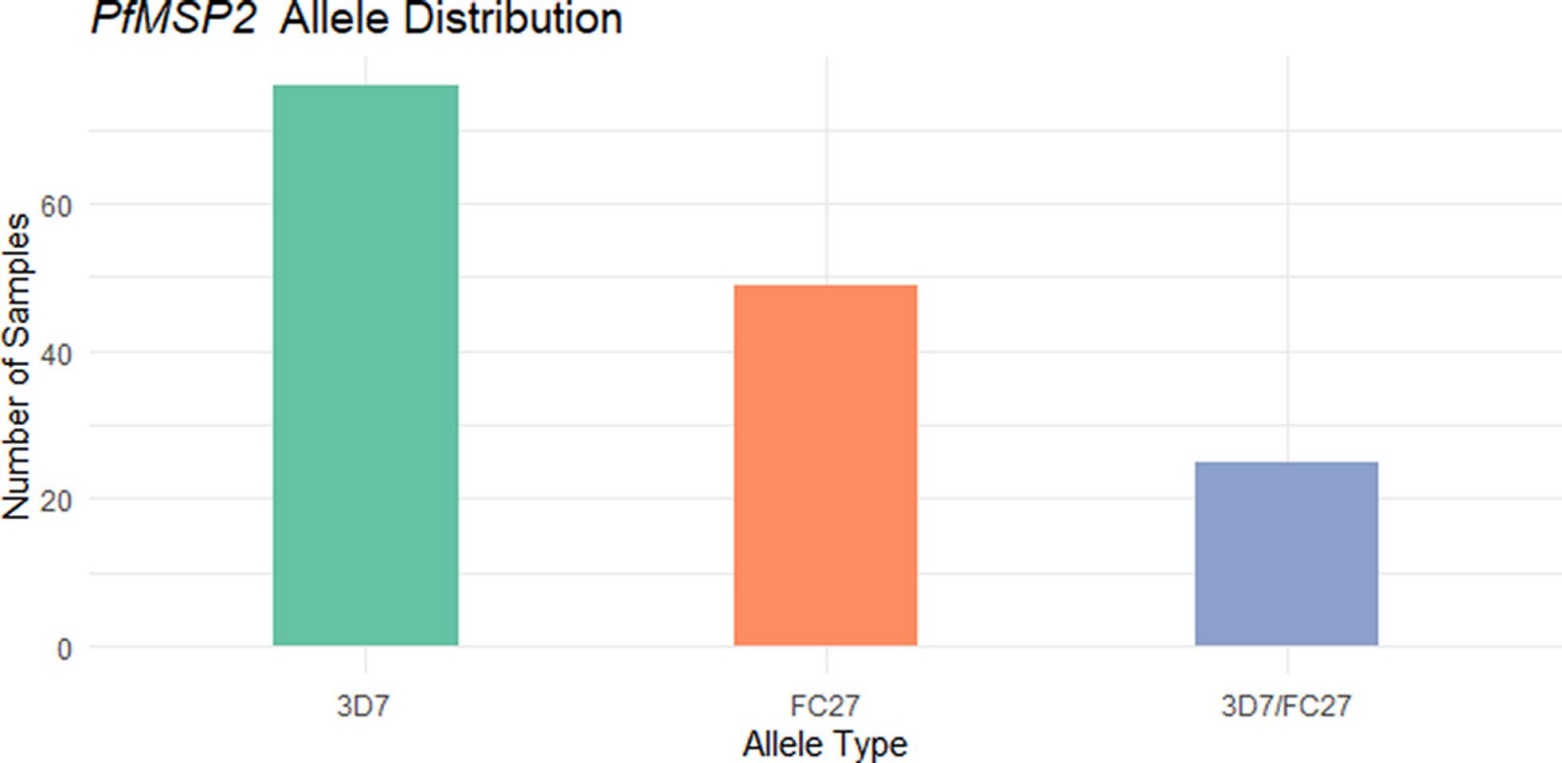
Distribution of *PfMSP2* allelic families. The 3D7 allele was the most common (56.3%), followed by FC27 (36.3%). Mixed infections with both alleles were detected in 18.5%

Of the 173 *P. falciparum*-positive samples, 168 were tested for *Pfmsp1* and *Pfmsp2*. Among the 168 genotyped samples, 35 (20.8%) were positive for *Pfmsp1* only, 10 (6.0%) were positive for *Pfmsp2* only, and 90 (53.6%) were positive for both *Pfmsp1* and *Pfmsp2*. Thirty-three samples (19.6%) failed amplification (Fig. 1). For *Pfmsp1*, the K1 allele was the most prevalent detected in 91 samples (67.4%), followed by RO33 in 42 samples (31.1%) and MAD20 in 33 samples (24.4%). All three alleles fragment size range was 150–300 bp. Mixed alleles were identified in about half of the successfully genotyped samples: K1/MAD20 in 16 samples (11.8%), K1/RO33 in 21 samples (15.5%), MAD20/RO33 in 10 samples (7.4%) and K1/MAD20/RO33 in 6 samples (4.4%) (Fig. 2). For *Pfmsp2*, the 3D7 allele was the most dominant detected in 76 samples (56.3%) (fragment size 200–500 bp), followed by FC27 in 49 samples (36.3%) (fragment size 350–500 bp). Co-occurrence of both alleles (3D7/FC27) was observed in 25 samples (18.5%) (Fig. 3).

Eighty samples (59.3%) were monoclonal and 55 (40.7%) were polyclonal (MOI > 1). The mean MOI was 1.5 [1.3–1.8], which was similar over the years (p = 0.7) (Table 1). The mean MOI among patients enrolled in rural health care settings (Sengerema) was similar to that in urban health care settings (Sekou Toure and BMC) (1.6 versus 1.4, p = 0.12).

**Table 1:** Yearly distribution of positive malaria samples and MOI in Mwanza, Tanzania

Year	Number of positive samples	MOI	Number of samples with NS mutation in *PfK13*	Amino acid changes in *PfK13* NS mutations
2016	31	1.32	1 (3.2%)	R529K
2017	46	1.78	3 (6.5%)	Y519D, A578T, A578S
2018	24	1.41	1 (4.2%)	A578S
2019	5	1.40	1 (20%)	A578S
2021	13	1.53	2 (15.4%)	G544E, A578S
2022	16	1.37	2 (12.5%)	R561H

Number of samples with *PfK13* NS mutations and corresponding amino acid changes

### *Pfk13* and *Pfmdr1*

*Pfk13* was successfully sequenced for 143 samples. Among these, 7.0% (10/143) exhibited non-synonymous mutations, including two cases of the validated AR marker R561H (1.4%). Other mutations were: Y519D, R529K, G544E, A578T, A578S, each occurring once, except for the latter occurring in 4/143 (2.8%). All mutations were detected as heterozygous signals except for two (561H and 578S) (Table 1). The two febrile patients carrying *P. falciparum* with *Pfk13* R561H both lived in the Kigoma region. The first patient, a 36-month-old male, was admitted in June 2022 at BMC. The second patient, a 12-month-old male, was admitted in July 2022 at BMC. *Pfmdr1* was successfully sequenced for 97 samples. All carried N86 (wild type), while 184 F was observed in 43.3% (42/97).

## Discussion

A high *Pfk13* mutation prevalence (7%) was found among paediatric malaria patients in Mwanza, Tanzania, and 1.4% carried the validated AR marker 561H. The *Pfmdr1* wild-type marker N86 was present at 100%, indicating an increased lumefantrine tolerance [23]. Multiplicity of infection was low, suggesting a low malaria transmission rate, and stayed similar over the years.

Tanzania is a high burden country for malaria, and young children are disproportionally affected. The global persistence of *P. falciparum* transmission despite control measures is attributed to the parasite’s genetic diversity enabling adaptation to antimalarials [26, 27]. Indeed, Tanzania is now one of the handful of countries in SSA where AR was confirmed [3]. Particularly the AR marker *Pfk13* 561H was found at high prevalence (20%) in northwestern Kagera in 2021, and the AR markers 622I and 675 V have also been reported [9, 28]. The 561H marker was detected in two patients presenting in 2022, both residing in Kigoma Region, approximately 600 km from the study site. Unfortunately, the information on the duration of stay, travel route, or reason for travel was not available for the two patients. Malaria has an incubation period of 7–30 days, but without knowing when the two children left Kigoma or how long they stayed in Mwanza before symptoms appeared and they were tested, it is not possible to determine whether the infection was contracted in Mwanza, Kigoma or somewhere along their travel route. This lack of information makes it impossible to determine whether the mutation was circulating in Mwanza 2022 or if it was imported to the city, which served as a distribution hub. Other non-synonymous *Pfk13* mutations included 578S in 2.8%, and, each once, 462 F, 473Y, 485R, 554 K, and 705D. Mutation 578S is frequently seen in SSA [29–32], but despite being located in the AR-relevant propeller domain of *Pfk13*, no link with antimalarial drug resistance has been identified as of today. The 554 K mutation has previously been reported in Senegal, but is of unknown relevance [31, 33]. The non-synonymous mutation prevalence reported in this study is higher compared to figures from elsewhere found in Tanzania, e.g., 0.4% found in a study conducted in 8 sites of Tanzania from 2016 to 2021 [28]. On the other hand, the 561H prevalence was low compared to findings from studies conducted in northwestern Tanzania in 2022 and 2023 [2, 34]. Mwanza is an economically bustling city and serves as major crossing point for travel routes in East Africa [35, 36]. The potential dilution of the *P. falciparum* parasite population by travellers may contribute to a delayed establishment of AR mutations that are successful elsewhere (such as *Pfk13* 561H). At the same time, population movement to and from Mwanza may facilitate the spread of resistant parasite lines within East Africa.

Regarding *Pfmdr1*, the N86 wild-type allele was present in all samples, while the 184 F allele was found in almost half. Both are associated with reduced lumefantrine susceptibility [37, 38]. Similar patterns are seen across East Africa [39].

A mean MOI of 1.5 was observed, which is lower than the mean MOI of 2.36 reported between 2017 and 2019 at a health centre in Igombe, a semi-urban area in Mwanza Region [40]. This might be due to the specific paediatric population included here, as MOI and age are inversely correlated [41]. Single-genotype *Pfmsp* infections of the K1 and 3D7 families were predominant, accounting for 67 and 56% of cases, respectively. This aligns with previous findings in Mwanza [40].

A major limitation of this study is the relatively small and inconsistent sample size across different years, with no samples collected in 2020 due to COVID-19 pandemic restrictions. Additionally, seasonality was not considered in the sample collection process and the study was conducted in a confined region, and the findings may not be generalizable. Samples were collected throughout the year, and although Mwanza is generally endemic for malaria, transmission intensity varies significantly within the region. The study sites were limited to health care facilities in semi-urban and peri-urban areas, where vector exposure is likely lower compared to rural areas. Moreover, potential biases in sample selection such as focusing primarily on symptomatic patients and excluding asymptomatic carriers, could affect the representativeness of the data. In addition, malaria control efforts in the region have intensified in recent years and might have contributed to reduce transmission. To provide context, a recent community-based study conducted in Kagera Region, north-western Tanzania, by Budodo et al*.* [42], reported a *P. falciparum* prevalence of 35.3% using qPCR among both symptomatic and asymptomatic individuals. However, their study employed a more sensitive diagnostic method (qPCR vs. conventional PCR in this study) and included a broader population base.

A further limitation is that the use of agarose gel electrophoresis for assessing *msp1* and *msp2* allelic families may introduce a bias in detection sensitivity and resolution [43]. Additional in this study other molecular markers associated with partner drug resistance, such as *Pfcrt*, *Pfmdr1* copy number, and *Pfplasmepsin 2/3* copy number were not assessed, due to limitations in DNA quantity and quality from dried blood spot samples. The samples were primarily analysed to investigate febrile illness in paediatric patients, with a focus on mosquito-borne pathogens (including arboviruses and *Plasmodium* spp). The remaining sample material was used to assess *P. falciparum* multiplicity of infection and key drug resistance markers. The present study focused on most relevant markers in the region: the *Pfk13* propeller domain, associated with partial artemisinin resistance, and *Pfmdr1* codon 86 and 184, given the widespread use of lumefantrine in Tanzania. Codon 1246 was excluded based on local data showing fixation on the wild-type allele since 2016 [28]. This is noted as an important limitation and the need to include these markers for molecular surveillance in the future is emphasized.

The follow-up studies, are designed to include larger, more diverse sample populations, to capture the dynamics of resistance markers over time and for which the presented data here serve as baseline. A strength of the study is the longitudinal follow up, and inclusion of MOI and genetic diversity data, which is sparse for SSA. The latter becomes increasingly important in this region of emerging AR because treatment efficacy studies may rely on MSP allelic diversity to distinguish reinfection from recrudescence.

## Conclusion

This study confirms the presence of a validated artemisinin resistance marker in the Mwanza region among paediatric patients in 2022. However, since the children came from Kigoma, it remains unclear whether the infection was contracted in Kigoma or Mwanza. Overall, AR markers are of low abundance, but the *PfMDR1* pattern indicates potential compromised lumefantrine sensitivity. The multiplicity of infection was found to be relatively low and stable compared to neighbouring regions. Although ACT is still highly efficacious in Tanzania, the present study demonstrates large regional differences in *P. falciparum* diversity and AR markers, advocating for continuous, integrated surveillance systems. 

## Data Availability

Data are available from the corresponding author upon reasonable request.

## Abbreviations

ACT: Artemisinin-based combination therapy
AR: Artemisinin partial resistance
ART: Artemisinin
BMC: Bugando Medical Centre
CUHAS: Catholic University of Health and Allied Sciences
DBS: Dried blood spot
DNA: Deoxyribonucleic acid
ELISA: Enzyme‐linked immunosorbent assay
MOI: Multiplicity of infection
PCR: Polymerase chain reaction
*Pfk13*: *Plasmodium* *falciparum kelch-13* Gene
*Pfmdr1*: *Plasmodium* *falciparum multidrug drug resistance 1* Gene
*Pfmsp*: *Plasmodium falciparum merozoite surface protein* Gene
*Pf*MSP1: *Plasmodium falciparum merozoite surface* Protein 1
*Pf*MSP2: *Plasmodium falciparum* Merozoite surface protein 2
RT-PCR: Reverse transcriptase‐polymerase chain reaction
SSA: Sub-Saharan Africa
WHO: World Health Organization
